# The Relationship Between Social Mobility Belief and Learning Engagement in Adolescents: The Role of Achievement Goal Orientation and Psychological Capital

**DOI:** 10.3389/fpsyg.2022.792108

**Published:** 2022-04-06

**Authors:** Jin Xie, Bo Zhang, Zhendong Yao, Biao Peng, Hong Chen, Juan Gao

**Affiliations:** ^1^School of Educational Sciences, Hunan Normal University, Changsha, China; ^2^Mental Health Service Center, Huanghuai University, Zhumadian, China; ^3^School of International Education, Huanghuai University, Zhumadian, China; ^4^School of Public Administration, Hunan Normal University, Changsha, China; ^5^School of Humanities and Management, Hunan University of Chinese Medicine, Changsha, China; ^6^Faculty of Health Management, Weifang Vocational College of Nursing, Weifang, China

**Keywords:** adolescent, social mobility beliefs, achievement goal orientation, psychological capital, learning engagement

## Abstract

**Objective:**

To explore the relationship between adolescents’ social mobility belief and their learning engagement, as well as the mediating effect of achievement goal orientation and the moderating effect of psychological capital.

**Method:**

A sample of 895 adolescents from Hunan, Jiangxi, Hainan, Henan, and Guangdong provinces were assessed using the social mobility belief questionnaire, the achievement goal orientation questionnaire, the adolescents’ psychological capital questionnaire, and the adolescents’ learning engagement questionnaire.

**Results:**

First, adolescents’ social mobility belief was positively related to their learning engagement (*r* = 0.481, *p* < 0.01); Second, the two achievement goal orientations-mastery goal orientations and performance goal orientations-had mediating effects between social mobility belief and learning engagement (*r* = 0.603, *p* < 0.01; *r* = *0.367*, *p* < 0.01); Third, the relationship between achievement goal orientation and learning engagement was regulated by psychological capital. Adolescents with high psychological capital had higher learning engagement (*r* = 0.684, *p* < 0.01).

**Conclusion:**

Adolescents’ social mobility belief can directly affect their learning engagement, and it can also indirectly affect their learning engagement through achievement goal orientation moderated by their level of psychological capital. Our findings highlighted the importance of providing adolescents with guidance in setting up positive social values and definitions of success while stimulating their psychological capital as a part of the teaching process.

## Introduction

Learning engagement refers to the levels of time and energy learners invest in their studies (Kuh, 2003). Learning engagement reflects the state of adolescents’ concentration abilities and studying conditions (Newman et al., 1992), and is an important indicator of academic achievements (Salanova et al., 2010; Zhang, 2012), learning process and development status (Liu, 2015), and even the quality of the learner’s school education. Exploring adolescents’ learning engagement and its mechanisms can help guide educational reforms to “academic challenge” and to promote deeper learning.

**FIGURE 1:**
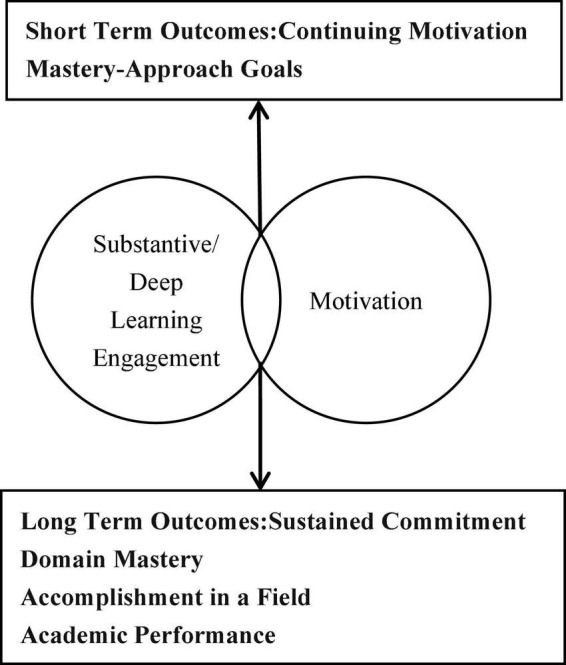
Conceptual model of the interaction between deep engagement and motivation, with associated short-term and long-term outcomes.

Learning engagement, in reflecting an individual’s attitude and behavior toward learning, is influenced by internal and external factors, such as motivation and environment. We argue that deep learning engagement is likely to be more strongly related to authentic motivational orientations and educational attitudes that are transferable into higher levels of academic performance (see Figure 1). When adolescents were substantively and deeply engaged with learning behavior, the psychological state was similar to that characterized as flow experiences (Christenson et al., 2012). Chapman (2002) divided learning engagement into cognitive elements, behavioral elements, and emotional elements. Fredricks et al. (2004) stated that learning engagement included behavioral engagement, cognitive engagement, and emotional engagement. Previous studies have explored numerous relationships which can impact learning engagement, such as student self-efficacy (Li and Bai, 2018), achievement goal orientation (Dweck, 1986), psychological capital (Ortega-Maldonado and Salanova, 2018; Sánchez Cardona et al., 2021), family socioeconomic status (Randolph et al., 2004; Jing-Jing et al., 2021), parents’ anticipation, the parent–child relationship (Newman et al., 1992), parenting styles (Alhadabi et al., 2019), in-class learning resources (Atmodiwirjo, 2013), and interpersonal relationships and support (Ferguson-Patrick, 2020). As an intrinsic motivational factor, researchers have more recently began to explore the relationship between social belief and learning engagement from the perspective of social cognition in recent years (Xie et al., 2018). Based on the literature, this paper borrows this model and argues that social mobility belief, as a kind of social cognitive tendency and an expectation of individual efforts to strive upward, can regulate individuals’ attitudes and behaviors by providing them with a way of thinking. Studies have found that there is a correlation between social mobility belief and learning engagement (Scherger and Savage, 2010), but few studies have thus far explored mechanism of this relationship. Therefore, the current study aimed to explore the relationship and mechanism between adolescents’ social mobility beliefs and their learning engagement.

Social mobility belief is an individual’s perception and judgment of objective social mobility, that is, one’s subjective judgment and expectations regarding whether they can achieve upward social mobility through their own efforts (An, 2015; Kostromina et al., 2017). Social stratification exists due different people possessing different social resources which then stimulate their expectation of change in one’s social status (social mobility) (Khakhulina and Tuček, 1997). The level of social mobility beliefs will affect individuals’ cognition and behavior. Individuals with higher expectations of mobility beliefs will study harder and strive for better social development (Xie et al., 2018). Studies also have shown that the effects of social mobility beliefs depend on the level of psychological capital (Morgan, 2018; Yang, 2018). For adolescents, achieving good results through learning engagement will help them enrich their own resources while providing them with opportunities for mobility between social classes to improve their social status (Breen, 2010; Pfeffer and Hertel, 2015). Empirical studies have also shown that higher levels of social mobility belief can enhance students’ perseverance in learning, improve their engagement with learning (Martin and Dowson, 2009), and increase their academic achievements (Day and Fiske, 2016). However, a lower belief in one’s social mobility also affects the degree of their learning engagement and pursuance of academic goals (Browman et al., 2017). Clearly, social mobility belief can be an important indicator in predicting learning engagement.

Achievement goal orientation refers to an individual’s intention or reason for an individual to strive to complete a task (Elliot et al., 2011; Maggio et al., 2016), and is an important internal dynamic factor for an individual. Elliot et al. (2011) have suggested that every individual has different abilities, and achievement goal orientation can be split into two groups: first, mastery goal orientation, which focuses on ability variability wherein the mastery of tasks and the development of one’s own abilities is the motivating goal, and second, performance goal orientation, which focuses on ability stability, wherein the individual focuses on performance ability and is motivated by a desire to avoid being seen as having low abilities (Nicholls, 1984). Different goal orientations will clearly lead to different school behaviors. Empirical research also shows that goal orientation is related to individual learning engagement (Robert et al., 2012), and that furthermore, different goal orientations have been shown to predict different levels of learning engagement in students (Walker et al., 2006).

With its impact on an individual’s expectation of their ability to realize upward social mobility, social mobility belief has a motivational effect on one’s attitude and behavior. Goal orientation and pursuit of goals can be regulated in individuals in order to achieve upward mobility to a higher social class (Kraus et al., 2013). Empirical research has also shown that social mobility beliefs in students can have influence their motivation to pursue success (Torres, 2015), their choices of goals (Shane and Heckhausen, 2013), and their individual efforts and behavior (Johnson et al., 2011; Kraus et al., 2013; Stephens and Townsend, 2013).

Achievement goal orientation is a motivation theory put forward by Dweck (1986) from the perspective of social cognition. The difference of individual’s implicit concept of intelligence reflects that there are two different learning motivations in learning: mastery goal orientation and performance goal orientation. Among them, students who master goal orientation think that the purpose of learning is to develop their own abilities. Performance-oriented students think that academic achievement is the proof of their ability, learning and learning is for social comparison and external reward (Dweck, 1986). Mastery goal orientation has a positive effect on learning, however, the impact of performance goal orientation on learning has presented inconsistent research results. On this basis, the researchers introduced the dimension of “approach-avoidance” into performance goal orientation, and formed a three-factor framework of achievement goal theory, namely mastery goal orientation, performance-approach goal orientation, and performance-avoidance goal orientation (Elliot and Church, 1997). The effects of mastery goal orientation and performance-avoidance orientation on academic achievement were more consistent. Specifically, master goal orientation has a promoting effect on learning, which is manifested in that individuals have higher learning emotions, tend to challenge difficult problems, and actively adopt learning strategies. Performance-avoidance orientation is detrimental to academic development, as students tend to adopt avoidant learning strategies (Lee and Kim, 2014).

Different goal orientation can lead to different behaviors. For adolescents, factors such as individual self-confidence, optimism, and judgment of self-ability will affect their choice of achievement goal orientation, which in turn affects their learning engagement. Furthermore, performance-goal-oriented adolescents tend to choose relatively easy learning tasks or to show their charisma. Adolescents who master goal orientation tend to acquire new knowledge and solve problems as goals, they believe that they are competent enough to solve the difficulties they encounter, so they are also willing to invest more time and energy in learning rather than choosing to avoid problems. But if they can’t recover from failure after repeated persistence, goal-oriented teenagers will consciously avoid tasks beyond their own abilities, because they will also consider their personal image when they realize that their abilities are inadequacies.

Psychological capital plays an important role in replenishing energy, stimulating motivation, maintaining work vitality and regulating individual attitudes and behaviors, and is an important part of individual dynamic system (Fredrickson, 2001). In addition to intellectual ability, physical ability and social resources, psychological capital is another extremely important factor that can affect individual cognition and action (Luthans et al., 2004). Generally, individuals with high psychological capital have abundant positive psychological resources and always face tasks with a positive attitude, especially when they encounter difficulties and setbacks in tasks, they see full of hope the future, optimistic in the face of temporary failure, perseverance, and never give up easily (Mao and Tan, 2015). Psychological capital refers to an individual’s positive state during the process of personal growth, and is an internal positive resource that both supplements their energy levels and motivates them to continue to grow (Luthans et al., 2006). Target orientation is an internal driving force, and the process of target selection and persistence is a process of energy consumption. According to the theory of resource conservation (Schaufeli and Bakker, 2004), psychological capital can provide one with resources to supplement their energy for target orientation, among other processes of energy consumption (Ahmadigatab et al., 2011). Studies have also shown that psychological capital can mediate individual achievement goal orientation. Adolescents with high levels in aspects of psychological capital (i.e., high self-efficacy, high resilience, and optimistic style) (So, 2008; Davari et al., 2012), strong academic responsibility (Wong, 1995) are more inclined to choose to master goal orientation. Therefore, it can be inferred that adolescents with a higher level of overall psychological capital and sufficient energy benefit from external support which allows or encourages them to devote themselves to learning to achieve their goals. That is to say, the higher the level of an adolescent’s psychological capital, the more important the role of social mobility belief is in promoting the adolescent’s learning engagement through achievement goal orientation.

To contribute to the existing understandings of the effects of social mobility belief, the current study put forward two hypotheses. Hypothesis 1: Adolescent achievement goal orientation plays a mediating effect on the relationship between social mobility belief and their learning engagement. Hypothesis 2: Adolescent psychological capital plays a mediating role in the relationship between achievement goal orientation and learning engagement. Hypothesis 3: Adolescent achievement goal orientation and psychological together play a moderating role in the relationship between social mobility belief and learning engagement.

## Materials and Methods

### Participants

The study participants were recruited from seven middle schools located in Henan, Hunan, Hainan, Guangdong, and Jiangxi provinces in China. A total of 895 students were recruited to complete the questionnaires. Participants were 15–18 years old (*M* = 17.50, SD = 1.87), 466 (52.0%) were boys and 429 (48.0%) were girls, 264 (29.5%) were in 9th grade, 276 (30.8%) were in 10th grade, 196 (21.9%) were in 11th grade, and 159 (17.8%) were in 12th grade. All the participants filled in the questionnaire through online.

### Measures

#### The Social Mobility Belief Questionnaire

The social mobility belief questionnaire (SMBQ) was compiled by Ming (2013) and measures six items (such as “As long as I keep working hard, I can improve my current situation” and “Compared with my parents’ generation, I will have a much higher social status in the future”) using a 6-point Likert-type scale from 1 (“completely inconsistent”) to 6 (“completely consistent”). The higher the total score, the stronger one’s belief in their own social mobility. In this study, the Cronbach’s alpha coefficient of the SMBQ was 0.856.

#### The Psychological Capital Questionnaire (Chinese Version)

The Psychological Capital Scale developed by Wang et al. (2011). Combining Chinese culture and the psychological characteristics of adolescents, the scale introduces the dimension of responsibility on the basis of the original self-confidence, hope, optimism and resilience, and modifies the items in it to make it suitable for measuring the psychological capital of adolescents. According to the results of previous interviews, adolescents basically understood hope and optimism as the same concept, so this study deleted the dimension of hope. Therefore, the revised psychological capital scale of this study has four dimensions: self-confidence, resilience, optimism, and responsibility. It consists of 15 items (such as “Even if things don’t go well at work or study, I won’t be discouraged” and “I always bounce back quickly from setbacks”) rated using a 7-point Likert-type scale from 1 (“completely inconsistent”) to 7 (“completely consistent”), and is divided into four dimensions: self-confidence, resilience, optimism, and responsibility. The higher the total score, the higher one’s level of psychological capital. In the present study, the internal consistency coefficient of the psychological capital questionnaire (PCQ) was 0.935.

#### The Achievement Goal Orientation Questionnaire

The achievement goal theory is devoted to the study of the dominant types of goals adopted by individuals in achievement situations and the analysis of cognitive, affective, and behavioral results arising therefrom. It is a motivation theory directly used to explain students’ achievement behaviors in educational situations. In a nutshell, achievement goal theory has two main contents: first, it describes the types of goal orientation that individuals may take in achievement situations; second, it analyzes the reasons why individuals have different goal orientations. There are different criteria for the division of achievement goal orientation. In this study, a division of mastery goal orientation and achievement goal orientation was used. Based on Elliot and Church’s achievement goal orientation questionnaire, the AGOQ was revised by Jiang (2004). The questionnaire has 16 items (such as “It is important for me to do better than others” and “I like the learning task that inspire our desire to explore in class, even if it is difficult to learn”) in total, each rated using a 5-point Likert-type scale ranging from 1 (“very inconsistent”) to 5 (“very consistent”). The items are divided into two aspects: mastery goal orientation and performance goal orientation. The higher the total score, the higher one’s level of achievement goal orientation. The Cronbach’s alpha coefficient of the AGOQ in the current study was 0.882. The internal consistency coefficients of the mastery goal orientation sub-scale and the achievement goal orientation sub-scale were 0.830 and 0.875, respectively.

#### The Learning Engagement Questionnaire (Chinese Version)

Based on Schaufeli’s Chinese version of Learning Engagement Questionnaire, the LEQ was revised by Fang et al. (2008). The questionnaire contains 17 items (such as “When I concentrate on my studies, I forget things around me” and “I feel confident and energetic when I’m engaged in my studies”) and uses a 7-point Likert-type scale to rank each item from 1 (“completely inconsistent”) to 7 (“completely consistent”) to measure three dimensions: vitality, dedication, and concentration. The higher the total score, the higher one’s level of learning engagement. In this study, the internal consistency coefficient of the scale was 0.887.

#### Reliability and Validity Test of Scales

The reliability and validity of the measurement scale were subsequently evaluated. Confirmatory factor analysis (CFA) was used to establish the internal validity of each construct. CFA showed that the modified model fit the data well: RMSEA = 0.053 < 0.08, GFI = 0.965 > 0.9, AGFI = 0.953 > 0.9, and NFI = 0.979 > 0.9. The Cronbach’s alpha values of each construct were all above 0.7, indicating acceptable reliability. Regarding convergent validity, it can be evaluated by average variance-extracted (AVE) and factor loadings (FLs). The values of AVE and FL of each construct were higher than 0.6, indicating an acceptable level of convergent validity (Table 1; Hair et al., 2019).

**TABLE 1:** Reliability and validity analysis.

Scales	Cronbach’s alpha	AVE	FL
SMBQ	0.856	0.782	0.648–0.879
PCQ-C	0.935	0.607	0.641–0.861
AGOQ	0.882	0.692	0.844–0.922
LEQ-C	0.887	0.691	0.845–0.921

### Data Analysis and Common Method Bias Testing

SPSS 24.0 and PROCESS 3.0 were used to complete the data analysis. The Harman single factor test was used for exploratory factor analysis (Podsakoff et al., 2003). The results show that there were 18 factors with characteristic roots greater than 1, with the first factor explaining 36.83% of the total variance. As this is less than 40% of the critical criteria, this indicates that there was no evidence of common method bias in this study.

## Results

### Descriptive Statistics and Correlation Analysis

The mean, standard deviation, and correlation matrix of each variable are shown in Table 2. The results show that there was a moderate positive correlation between social mobility belief and learning engagement. Social mobility belief was positively correlated with mastery goal orientation, performance goal orientation, and psychological capital. Mastering goal orientation, performance goal orientation, and psychological capital were all positively correlated with learning engagement. Mastering goal orientation, performance goal orientation, and psychological capital also showed a significant positive correlation.

**TABLE 2:** Descriptive statistics and correlation analysis results of variables.

	*M* ± SD	Social mobility belief	Mastery goal orientation	Performance goal orientation	Psycho- logical capital
Social mobility belief	4.05 ± 0.93				
Mastery goal orientation	3.79 ± 0.68	0.418**			
Performance goal orientation	3.41 ± 0.65	0.377**	0.425**		
Psychological capital	5.15 ± 0.89	0.515**	0.631**	0.326**	
Learning engagement	5.21 ± 1.05	0.481**	0.603**	0.367**	0.684**

**p < 0.01.

### Testing Mediating Effect of Achievement Goal Orientation and Moderating Effect of Psychological Capital

**FIGURE 2:**
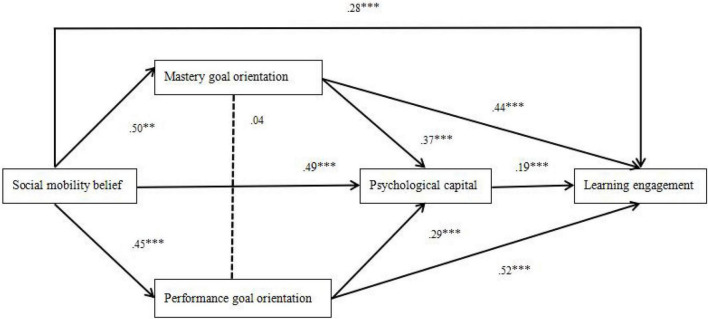
The mediating effect of psychological capital and achievement goal orientation between social mobility and learning engagement. ^**^p < 0.01, ^***^p < 0.001.

The test of the mediating effect of achievement goal orientation was done using Model 15 of the SPSS macro program PROCESS, as compiled by Hayes (2013; see Table 3). The bootstrap test showed that the mediating effect of mastery goal orientation was significant, with the 95% confidence interval being [0.16, 0.26] and the mediating effect being 0.20, which was 41.67% of the total effect (0.48). The mediating effect of performance goal orientation was also significant, with the 95% confidence interval of [0.05, 0.12] and a mediating effect of 0.08, which was 16.67% of the total effect (0.48). As shown in Figure 2, social mobility belief had a positive effect on psychological capital (β = 0.49, *p* < 0.001), and mastery goal orientation (β = 0.50, *p* < 0.001), and performance goal orientation (β = 0.45, *p* < 0.001) and learning engagement (β = 0.28, *p* < 0.001). Psychological capital had a positive effect on learning engagement (β = 0.19, *p* < 0.001). Mastery goal orientation had a positive effect on psychological capital (β = 0.37, *p* < 0.001) and learning engagement (β = 0.44, *p* < 0.001). Performance goal orientation had a positive effect on psychological capital (β = 0.29, *p* < 0.001) and learning engagement (β = 0.52, *p* < 0.001).

**TABLE 3:** The mediating effect of achievement goal orientation and psychological capital.

Result variable	Predictor variable	*R* ^2^	*F*	β	SE	*T*
Mastery goal orientation	Social mobility belief	0.16	191.02**	0.40	0.01	13.80**
Performance goal orientation	Social mobility belief	0.12	149.91**	0.36	0.01	12.22**
Learning engagement	Social mobility belief	0.41	336.13**	0.26	0.01	9.98**
	Mastery goal orientation			0.47	0.01	17.49**
Learning engagement	Social mobility belief	0.28	168.45**	0.38	0.01	12.99**
	Performance goal orientation			0.20	0.01	7.03**
Psychological capital	Social mobility belief	0.20	170.54**	0.48	0.01	13.45**
	Learning engagement			0.30	0.01	5.06**

**p < 0.01. All variables in the model were standardized and then put into the regression equation.

To test the moderating effect of psychological capital on the mediating role of different variables of achievement goal orientations, we performed a regression analysis using Model 15 (see Table 4). The results showed that the interaction between mastery goal orientation and psychological capital significantly positively predicted learning engagement (β = 0.03, *p* < 0.01), and the mediating effect of a high level of psychological capital on mastery goal orientation was significant, with an effect value of 0.14. The mediating effect of a low level of psychological capital on mastery goal orientation was also significant, with an effect value of 0.09. These results show that psychological capital plays a significant moderating role in this mediating effect. The mediating of high level of psychological capital on learning engagement was significant, with effect value of 0.15, the mediating of low level of psychological capital on learning engagement was also significant, with effect value of 0.13. To better interpret the moderating effect of psychological capital, psychological capital was divided into high and low groups (according to the average plus or minus one standard deviation). A simple slope test showed that the level of learning engagement in adolescents with low psychological capital improved as their level of mastery goal orientation increased (β = 0.20, *p* < 0.01). In adolescents with high psychological capital, this increase in level of learning engagement was even more pronounced (β = 0.30, *p* < 0.01).

**TABLE 4:** The moderating effect of psychological capital on mediating effects.

Result variable	Moderating effect of psychological capital				Criterion of judgment		
		β	SE	95% CI	INDEX	SE	95% CI
Learning engagement-mastery	High psychological capital	0.30	0.03	[0.26, 0.39]	0.022	0.01	[0.006, 0.042]
	Low psychological capital	0.20	0.03	[0.16, 0.28]			
	Mastery goal orientation × psychological capital	0.03	0.01	[0.03, 0.10]			
Learning engagement-performance	High psychological capital	0.15	0.03	[0.12, 0.23]	0.017	0.01	[0.002, 0.036]
	Low psychological capital	0.06	0.03	[0.02, 0.14]			
	Performance goal orientation × psychological capital	0.026	0.01	[0.02, 0.07]			

Regression analysis showed that the interaction between performance goal orientation and psychological capital also positively predicted learning engagement (β = 0.026, *p* < 0.01), and that a high level of psychological capital had a significant mediating effect on achievement goal orientation, with an effect value of 0.07. The mediating effect of a low level of psychological capital on achievement goal orientation was not significant. The simple slope test on the mediating effect of performance goal orientation showed that learning engagement in those with a low level of psychological capital increased with the improvement of performance goal orientation (β = 0.06, *p* < 0.01); in those with a high level of psychological capital, the level of learning engagement showed an even stronger increase (β = 0.15, *p* < 0.01).

## Discussion

The current study explored the relationship between social mobility beliefs and adolescents’ learning engagement, specifically its mechanism and boundary conditions. The results show that social mobility beliefs are correlated with learning engagement in that they can positively predict learning engagement. This finding is similar to previous research results that indicate that social mobility beliefs are related to learning attitudes and behaviors (Wesely, 2012; Atroszko, 2013). The results support Hypothesis 1 of the study, that social mobility beliefs are related to learning engagement. The results of the study support Hypothesis 3, achievement goal orientation and psychological capital play a moderating role in the relationship between social mobility belief and learning engagement. They also indicate that an adolescent’s expectation of social mobility (i.e., social mobility belief) has a motivational effect and can promote increased learning engagement.

The current study found that social mobility belief significantly positively predicted achievement goal orientation and positively predicted learning engagement through the mediating role of achievement goal orientation. These findings expand existing research on the correlation between achievement goal orientation and learning engagement (Christenson et al., 2012) and also confirm that social mobility belief, as a driving factor of learning engagement (Harris, 2008), can further affect an individual’s learning engagement by influencing their goal-setting and the motivational effects of pursuing their goals.

The results of the current study also show that the positive predictive effect of adolescents’ social mobility belief on mastery goal orientation (β = 0.42) is greater than that of performance goal orientation (β = 0.38). That is to say, the motivating effect of social mobility belief differs in its “motivational force” when individuals face their specific goals. This may be because the motive effect of social mobility belief stems from the acquisition of capital to achieve “social mobility.” The results suggests that the role of social mobility belief in goal orientation should be related to adolescents’ active engagement in learning activities. According to achievement goal theory, different achievement goal orientations produce different behaviors which can then have different influences on learning results. An individual with a mastery goal orientation believes that ability is changeable, and that goals can be achieved through hard work. They therefore tend to perceive social mobility as being something which they can control, and are more active in learning, which then has a relatively high impact on their learning engagement. However, an individual with an performance goal orientation has a defensive attitude toward ability, and the influence of social mobility expectation on learning engagement becomes more complicated. Previous studies have shown that mastery goal orientation positively correlates with adolescent academic achievement, but the prediction direction and effect of performance goal orientation are not clear (Harackiewicz et al., 1998; Pintrich, 1999).

The results of the current study show that psychological capital can play a moderating role in the second half of the mediating pathway as follows: adolescent social mobility belief → achievement goal orientation → learning engagement. This effect is even more obvious in those with an performance goal orientation. That is to say, when adolescents’ level of psychological capital is high, their social mobility beliefs play a strong role in their learning engagement through goal orientation. When their level of psychological capital is low, however, the effect of social mobility belief on learning engagement through performance goal orientation is not significant. This may be because psychological capital can supplement physical and mental energy for the process of goal-oriented energy exhaustion (Basinska and Rozkwitalska, 2020), meaning that the higher one’s level of psychological capital, the more it can provide sufficient compensation for the consumption of energy used in the process of achieving a goal, thus ensuring the individual’s motivation to continue to work hard toward the goal, and thus contributing further energy to the current learning tasks. Meanwhile, for those with a low sense of psychological capital, performance goal orientation consumes more resources to maintain their internal perception and external image of being “capable” and giving them a sense of security. The results of the current study show that the influence of social mobility belief on adolescents’ learning engagement through goal orientation, particularly in those with a performance goal orientation, is moderated by psychological capital, and that one’s level of psychological capital can further act as a boundary condition to their learning engagement.

### Implications and Suggestions

This study found that social mobility belief can significantly positively predict achievement goal orientation and positively predict learning engagement through the mediation of achievement goal orientation. The findings highlight the importance of encouraging adolescents to dismantle deep-rooted perceptions of class immobility through their education. First, the importance of the role of school education should be emphasized in order to improve adolescents’ willingness to receive and engage with learning. The propaganda function of school education should be considered, and used to actively promote the role of school education in improving students’ belief in social mobility, and to change negative views toward learning such as “studying hard is useless in my social context” and that knowledge and theory holds little value in their daily life. Second, teachers should guide adolescents in setting reasonable learning goals and help them manage learning difficulties by actively providing them with appropriate help. Adolescents can be taught how to formulate reasonable and clear learning objectives with consideration of learning difficulties in order to be able to complete tasks that are within their own abilities, which will then lead them to choose appropriate achievement goals in the future, reducing unnecessary struggle or disincentive to try hard. At the same time, appropriate situations and contexts should be provided to help adolescents gain successful experiences and enhance their self-confidence. Third, “compensatory education” should be made accessible to assist adolescents who were born into disadvantaged positions. Schools should play a leading and proactive role in this, by offering reduced education costs, increasing their overall investment in education, reworking poor education methods, and improving the quality of education they provide, among other things. By creating access to compensatory education, disadvantaged adolescents will have improved opportunities to receive a better school education, leading to a better and more equitable distribution of educational resources, thus promoting the upward mobility of their social strata – particularly those on the bottom – while optimizing social structures and promoting better social development.

The results of the current study also show that when adolescent psychological capital level is high, their belief in social mobility plays a strong role in learning engagement through goal orientation. However, when one’s level of psychological capital is low, the effect of social mobility belief on learning engagement through achievement goal orientation is not significant. Adolescent psychological capital therefore has an important influence on learning engagement. The current findings suggest that when working with adolescents – both individually or as together as in classes – it is important to help them develop their psychological capital to positively influence their social mobility belief to then influence their learning engagement. This can be done in a few ways. First, school education should not only emphasize developing knowledge and technical skills, but also pay attention to assisting students in developing their psychological capital. When the drive to master knowledge and skills has been generated and transformed into motivation, adolescents will take self-directed action to increase their investment in learning. Second, the inclusion of mental health courses within standard education will also help improve adolescents’ psychological capital by teaching them how to pay attention to their mental health and learn how to master appropriate coping strategies. Furthermore, such curriculum would provide adolescents with positive psychological suggestions which would enhance their self-confidence, building their optimism while teaching them how to interpret success or failure in a positive manner, and how to deal with setbacks constructively. Finally, adolescents should be encouraged to participate proactively in a variety of social activities to benefit to their psychological growth, allowing them to recognize their own areas for self-improvement and teaching them persistence in the face of difficulties.

### Limitations and Future Directions

The current study contributes to the current literature on the relationships between adolescent social mobility belief, learning engagement, and school education. However, there remain some shortcomings. Future research into adolescent social mobility belief and psychological capital could focus on a few aspects. First, when discussing the relationship between adolescent social mobility belief and learning engagement, the current study emphasized the importance of psychological capital, but did not deeply explore the factors which affect adolescents’ psychological capital. Future research could focus on the factors which affect adolescents’ psychological capital so as to expand the scope of application of the research results. Second, the sample in the current study was made up primarily of adolescents attending one of in five senior high schools or two junior high schools in Hunan, Henan, Hainan, Guangdong, and Jiangxi provinces in China, and the sample sizes taken from in Guangdong and Jiangxi provinces were relatively small. Therefore, the current findings cannot be generalized widely. Future research could expand the sample size, and carry out a comparative study with adolescents in other parts of the world to further verify the conclusions. Finally, the current study used a cross-sectional research method and was not a long-term longitudinal study. Future research could use a long-term follow-up research method, supplemented by case interview research, so as to improve the detail and applicability of the research results.

## Data Availability Statement

The original contributions presented in the study are included in the article/supplementary material, further inquiries can be directed to the corresponding authors.

## Author Contributions

JX and HC designed the study protocol. JX performed the statistical analysis and drafted the manuscript. BP guided the statistical analysis and interpretation of the results, edited the final manuscript, completed the literature review, and participated in the study design and interpretation analysis. ZY provided the financial support, as well as guided the first draft of the manuscript. BZ and JG provided the guidance on the overall design of the study and the revision of the manuscript. All authors contributed, read, and approved the final manuscript.

## Conflict of Interest

The authors declare that the research was conducted in the absence of any commercial or financial relationships that could be construed as a potential conflict of interest.

## Publisher’s Note

All claims expressed in this article are solely those of the authors and do not necessarily represent those of their affiliated organizations, or those of the publisher, the editors and the reviewers. Any product that may be evaluated in this article, or claim that may be made by its manufacturer, is not guaranteed or endorsed by the publisher.
